# Supplementary material for: Feasibility of gel-like radiopaque embolic material using gelatin sponge and contrast agent for tract embolization after percutaneous treatment
Source: PLoS One. 2023 Feb 3;18(2):e0281384. doi: 10.1371/journal.pone.0281384 (PMC9897536; doi:10.1371/journal.pone.0281384)
Supplement: S1 Table — (DOCX) [file pone.0281384.s001.docx]

| Characteristic | Value |
| --- | --- |
| No. of patients (M/F) | 12 (6/6) |
| Age, years, mean ± SD | 64.8 ± 12.7 |
| Pre-existing diseases  Cholangiocellular carcinoma  Liver metastasis  Gastric varix  Esophageal varix  Rectal varix  Gallbladder cancer  Portal vein thrombosis  Hepatocellular carcinoma  Other | 1  1  4  1  0  1  1  0  3 |
| Child-Pugh classification  A / B / C | 4 / 7 / 1 |
| Treatment  PTPE  PTS  Portal stenting  Other | 2*  5  2  3 |
| Portal vein for percutaneous access route  Right portal vein  Anterior branch of right portal vein  Posterior branch of right portal vein  Left portal vein | 1  3  1  7 |
| Sheath size  4-Fr  5-Fr  6-Fr  7-Fr | 9  2  1  0 |
| Platelet count, ×10^4^/µL, mean ± SD | 14.5 ± 10.2 |
| Hospital stay, days, mean ± SD (range) | 7.4 ± 3.9 (2–16) |
| Adverse Events  ^†^Postoperative hemorrhage  ^†^Portal vein thrombosis | N/A  N/A |

PTPE, percutaneous transportal embolization; PTS, percutaneous transhepatic sclerotherapy; SD, standard deviation.

* Ipsilateral puncture in 2 cases.

**^†^** As graded by Common Terminology Criteria for Adverse Events (CTCAE) version 5.0. Postoperative hemorrhage and portal vein thrombosis could not be evaluated due to no CT images during the follow-up period.
