# Supplementary figures and images for: Feasibility of gel-like radiopaque embolic material using gelatin sponge and contrast agent for tract embolization after percutaneous treatment
Source: PLoS One. 2023 Feb 3;18(2):e0281384. doi: 10.1371/journal.pone.0281384 (PMC9897536; doi:10.1371/journal.pone.0281384)

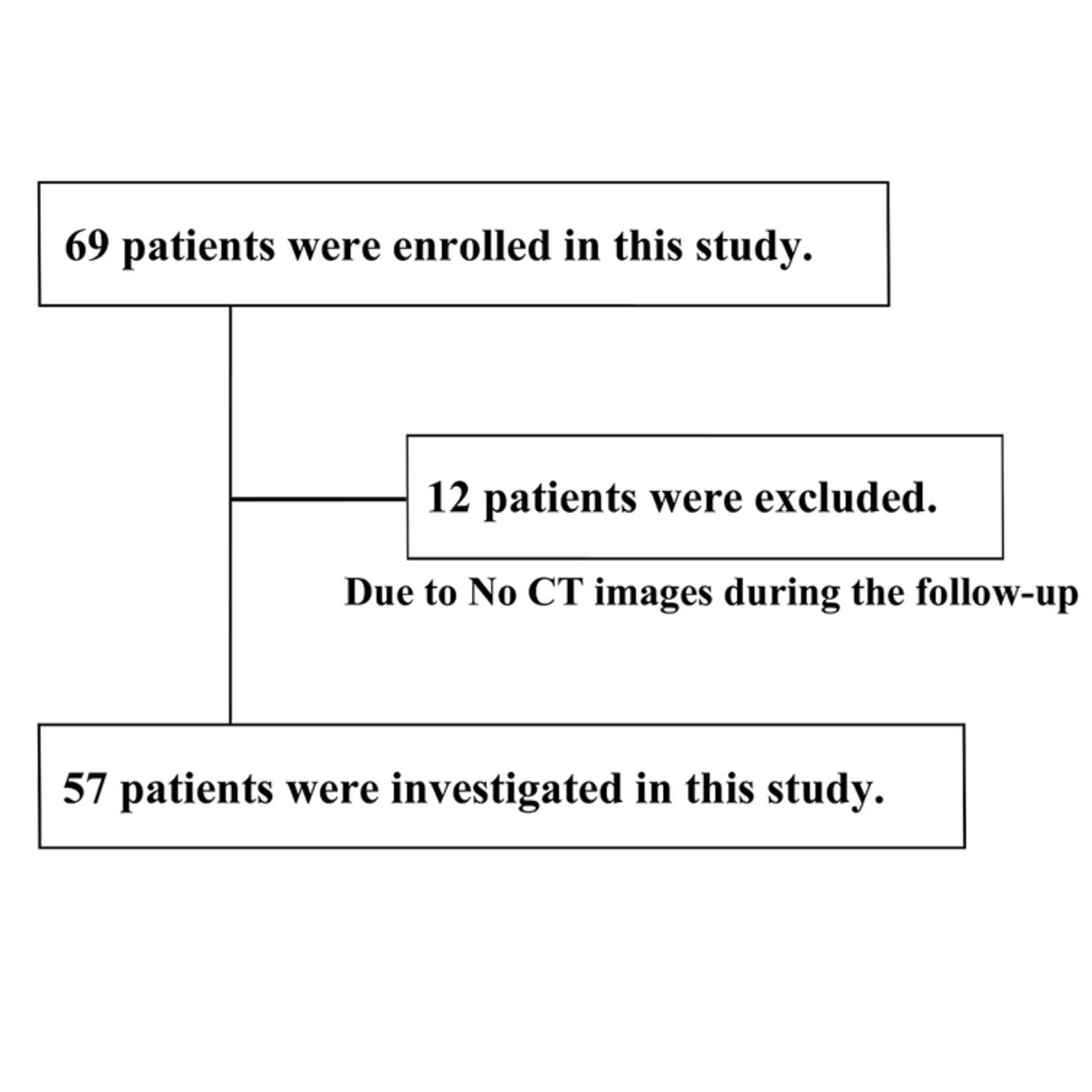

Supplement: S1 Fig — (TIF) [file pone.0281384.s002.tif]

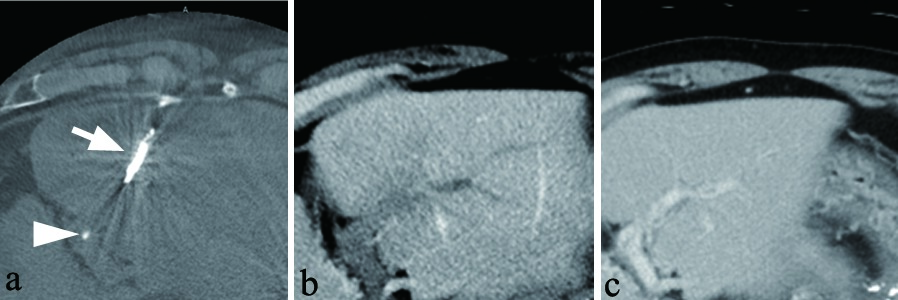

Supplement: S2 Fig — a) Post-embolization cone-beam CT showed spotty hyper-attenuation in the umbilical portion of the portal vein (white arrowhead) away from the embolized transhepatic tract (white arrow). b) Contrast-enhanced CT three days after the procedure did not visualize the left anterior branch or umbilical portion of the portal vein. c) Follow-up CT showed visualization of the left anterior branch and umbilical portion after anticoagulation therapy for three months. (TIF) [file pone.0281384.s003.tif]
