# Supplementary material for: Feasibility of gel-like radiopaque embolic material using gelatin sponge and contrast agent for tract embolization after percutaneous treatment
Source: PLoS One. 2023 Feb 3;18(2):e0281384. doi: 10.1371/journal.pone.0281384 (PMC9897536; doi:10.1371/journal.pone.0281384)
Supplement: Attachment — Submitted filename: ResponseToReviewers.doc [file pone.0281384.s006.doc]

**Dear editors and reviewers:**

Thank you for giving us this opportunity to submit a revised version of our manuscript. We appreciate the comments from the reviewers, which have enabled us to greatly improve our manuscript. We have modified the manuscript based on the helpful comments provided. We have added the requested material to the revised version of the manuscript as concisely as possible. We believe that the revised manuscript will be of interest to the readers of *PLOS One*.

**Reviewer #1:**

Thank you for your kind consideration of our manuscript. We are grateful for your comments, which have helped us to improve our manuscript. Please find below our point-by-point responses to the comments provided.

**Introduction**

**R-1. I miss the mention of percutaneous transhepatic biliary interventions, e.g. percutaneous transhepatic biliary drainage (PTBD), in the first paragraph.**

**Response:** Thank you for pointing this out. Based on your comment, we have revised the Introduction section as follows.

(Page 3, Line 49 - 51)

percutaneous transportal embolization (PTPE), percutaneous transhepatic biliary drainage (PTBD), and percutaneous transhepatic sclerotherapy (PTS) for esophagogastric varices [1-4]

We have also added Reference #4 in the revised manuscript.

Reference #4.

Covey AM, Brown KT. Percutaneous transhepatic biliary drainage. Tech Vasc Interv Radiol. 2008; 11:14-20.

**R-2. I consider the sentence “The hemostatic effect of embolic materials requires evaluation in animal experiments.” in line 72 to be too strict and not necessary.**

**Response:** We have revised the Introduction section based on this comment.

(Page 4, Line 71 - 72)

~~requires evaluation~~can be evaluated

(Page 4, Line 72)

prior to clinical application

**R-3. In my opinion, the introduction is too lengthy and should be streamlined. For example, the information given in lines 55 – 56 is negligible.**

**Response:** We have removed the sentence in question and have simplified the second paragraph of the Introduction.

(Page 4, Line 53 - 54)

~~In liver biopsy, 14- to 18-G needles are available for puncture (5,6). With PTPE and PTS, a 4- to 10-Fr sheath or catheter is generally inserted into the liver (2,3,7).~~

**R-4. Line 57 – 58: “However, these procedures carry a risk of accidentally puncturing structures such as arteries, portal veins, and veins, which would result in haemorrhage”. I find this sentence confusing, since in the setting of the presented procedures the underlying approach is the intentional creation of a transportal access. It would be appropriate if percutaneous biopsies were involved.**

**Response:** Thank you for pointing out that this sentence might be confusing to readers. To avoid misinterpretation, we have emphasized here that puncture procedures can potentially cause iatrogenic bleeding, and have removed descriptions of the specific types of blood vessels.

(Page 3, Line 54 – Page 4, Line 58)

However, ~~these procedures carry a risk of accidentally puncturing structures such as arteries, portal veins, and veins, which would result in hemorrhage (8,9)~~ these puncture procedures carry a risk of causing tract bleeding after removal of the puncture needle or sheath [6,7].

**Methods**

**R-5. Considering the retrospective study setting, I was surprised that non-enhanced CT was performed immediately after the procedure in all of the included cases. Is this regarded the internal standard in your department? Was the imaging of the embolic agent distribution the only indication for this examination? This aspect might be discussed in the context of radiation safety.**

**Response:** Thank you for raising this pertinent point. A hybrid CT/angiography system is available in the angiography room of our facility. As a result, CT was routinely performed to detect post-procedure hemorrhage just after tract embolization. Non-enhanced CT in the angiography room enables immediate evaluation of puncture tract bleeding such as perihepatic hematoma and intraperitoneal hemorrhage, as well as confirmation of the distribution of gel-like embolic materials.

**R-6. Was exactly 3 ml of the embolic material always used? I would expect the amount to depend on the length and size of the intrahepatic catheter tract.**

**Response:** Three milliliters of contrast agent and two sheets of absorbable gelatin sponge were consistently used for tract embolization. Three milliliters of contrast agent represents the minimum volume required to form gel-like radiopaque embolic material according to our basic experiments. Two sheets of absorbable gelatin sponge were the minimum required to pass through the internal cavity of the long sheath and to reach the tract in the liver.

**R-7. Line 136 – 138: “Non-enhanced CT performed immediately after the procedure identified no migration of embolic material from the tract and no hyperdense fluid accumulation surrounding the liver.“**

**This paragraph should rather be moved to the results section.**

**Response:** Thank you for pointing this out. We have deleted this sentence from the Methods section and moved it to the Results section.

(Page 10, Line 138 – 139)

~~identified no migration of embolic material from the tract and no hyperdense fluid accumulation surrounding the liver.~~

(Page 15, Line 227 – 232)

~~An area of high concentration in the hepatic tract was confirmed on CT immediately after embolization. No bleeding derived from the hepatic tract was evident on CT (Fig 1C). No migration of embolic material was observed from the hepatic tract on CT (Fig 1C)~~ Non-enhanced CT identified no migration of embolic material from the tract and no accumulation of hyperdense fluid surrounding the liver (Fig 1C).

**R-8. Animal experiment: How many punctures were performed per animal? Based on Figure 4, I would expect that six attempts were made per embolization material? Was only one type of embolic material used in the same animal? You should provide more information on this.**

**Response:** Thank you for pointing this out. We agree with your interpretations. We have added the following sentences to provide more information.

(Page 13, Line 194 - 195)

A total of six punctures per animal was performed, with each kidney punctured three times.

(Page 13, Line 201 - 202)

Each embolic material was used for tract embolization in one rabbit.

**R-9. Furthermore, the extent of bleeding may depend on the location of the puncture tract. A central puncture at the level of the renal hilus is more likely to lead to a relevant bleeding than a puncture at the upper or lower peripheral organ pole. If I understand correctly, the kidney of the animals were punctured blindly. Thus, there was no control of whether a larger vessel was punctured or not. How could it be ensured that the bleeding severity was comparable for each puncture? That aspect might be also worth mentioning in the limitation section.**

**Response:** Thank you for raising this issue. We performed the puncture radially to the renal hilum. The kidney received intravenous injection of contrast agent and was viewed under X-ray fluoroscopy before puncture. This contrast method allowed visualization of a clear outline of the kidney. Figure 3 shows a kidney after intravenous injection of contrast agent. The following sentence was added to the Methods section of the revised manuscript.

(Page 13 Line 195 - 196)

Each puncture was performed radially in the direction of the renal hilum under X-ray fluoroscopy.

**R-10. I don´t quite understand why the kidneys were i.v. contrasted when they were surgically exposed for the puncture anyway.**

**Response:** Thank you for this comment. The kidney received injection of contrast medium because the shape of the organ needed to be visualized under X-ray fluoroscopy during tract embolization. This enabled puncture in the direction of the renal hilum once the shape of the contrast-enhanced kidney was understood. In addition, we assumed that the contrast-enhanced image of the kidney in Figure 3 might help readers to understand the procedure of tract embolization in this animal experiment. We have also explained our procedure to enhance reader comprehension. Please see the legend for Figure 3.

(Page 19 Line 292 - 294)

The kidney was intravenously injected with contrast agent to allow operator understanding of the outline of the kidney.

**R-11. Why was the pathological analysis of the embolized kidneys only carried out with the gel-like embolic material? The cases after coil embolization may be difficult to process as serial sections, but those after NBCA embolization could have been interesting, especially in comparison to the gel-like embolic material.**

**Response:** Thank you for this interesting comment. In the present study, we did not compare results from pathological evaluation of the embolized tract with those of other embolic materials. This is because making pathological slides for the coil embolization arm is difficult. We focused on making pathological slides for the gel-like embolic material arm. As you mentioned, evaluating renal tracts after NBCA embolization in comparison with those after embolization with gel-like embolic material might be interesting. Pathological phase of the study would be required in near future.

**R-12. What was the embolic agent of your choice before the described gel-like material was introduced?**

**Response:** Tract embolization using embolic material with gelatin sponge has been performed at our facility since 2000. Collecting information on medical records prior to December 2006 was difficult, because the paper medical records were out of date. According to our investigations, from January 2007 to December 2013, a total of 39 consecutive cases underwent tract embolization after transhepatic puncture procedure. In all cases, a gel-like embolic material with gelatin sponge and contrast media was used for tract embolization via the transhepatic puncture route. In the previous series, the gel-like embolic material comprised two sheets of gelatin sponge and 4–6 mL of contrast agent. We did not have specific protocols at that time regarding the volume of contrast agent. The previous method used a larger volume of contrast medium than the current method. Two of the 39 cases (5.1%) needed emergent TAE after post-procedural life-threatening bleeding, classified as Grade 4 (severe) according to CTCAE version 5.0. Six cases showed minor complications, such as perihepatic hematoma and biliary hemorrhage that were detected on CT immediately after the procedure and did not require blood transfusion during hospitalization. In those six cases, follow-up CT was performed within 30 days after the procedure to confirm hematoma regression. All six cases were classified as showing Grade 1. We assumed that the bleeding events occurred because the amount of contrast agent was larger than that in the current study. We therefore reduced the amount of contrast agent to make the embolic material more viscous than in the previous method. The present study used gel-like embolic material comprising two sheets of gelatin sponge and 3 mL of contrast agent.

**Results**

**R-13. In the cases of non-target embolization, what was the percutaneous access route? Do the authors believe the access route might potentially influence the occurrence of such complications?**

**Response:** In the two case of non-target embolization, the access route was the left branch of the portal vein. However, we do not believe that the left branch of the portal vein is more associated with non-target embolization compared to the right branch of the portal vein. Non-target embolization just occurs when the tip of the sheath remains in the portal vein. The tip of the sheath should be withdrawn from the portal vein. We could see the tip of the sheath under X-ray in all 57 cases when we started tract embolization. Unfortunately, we placed the tip of the sheath in the wrong position in the two cases in question.

**R-14. The authors describe two cases of subcutaneous hematomas at the puncture site. Did non-enhanced post-procedural CT show no perihepatic fluid accumulation in these cases either?**

**Response:** In both cases of subcutaneous hematoma at the puncture site, no perihepatic hematoma was observed on non-enhanced CT after the procedure.

**Discussion**

**R-15. I think the discussion is quite lengthy and could be condensed.**

**Response:** Thank you for raising this issue. We have attempted to shorten or remove sentences in the Discussion section to improve text flow and make the section more concise.

(Page 21, Line 332 - 334)

~~These embolic materials are difficult to see under X-ray fluoroscopy, and~~ Clinicians cannot confirm tract hemostasis with such radiolucent embolic materials.

We would like to ask you to confirm the other deleted sentences provided in the R-16, 18 and 20.

**R-16. Since the presented study deals with percutaneous tract embolization after transportal interventions, I think some of the literature cited in line 306 – 311 is not suitable. On the other hand, I miss literature that explicitly deals with the embolization of access tract after percutaneous transportal procedures, such as:**

**- Embolization of the Transhepatic Tract after Percutaneous Portal Vein Interventions: Single-Centre Retrospective Study Comparing n-butyl Cyanoacrylate Versus Coils by Zi-Han Zhang et al.**

**- Feasibility of Mynxgrip®-Assisted Percutaneous Transhepatic Portal Venous Access Closure, by Gary X V Tan et al.**

**- Gelfoam for Closure of Large Percutaneous Transhepatic and Transsplenic Puncture Tracts in Pediatric Patients by Uller et al.**

**Response:** Thank you for these helpful comments and for providing these references. As suggested, we have deleted the sentences in question and have added new references to revise the paragraph as follows.

(Page 21, Line 322 - 330)

~~As an embolic material with gelatin sponge, Chuang et al. used absorbable gelatin sponge particles for tract embolization of liver biopsy tracts to control bleeding (19). Smith et al. reported a useful method for injecting a gelatin sponge torpedo into the liver tract after transhepatic liver biopsy~~ ~~(6). Tran et al. (20) and Reiner et al. (21) demonstrated a gelatin sponge slurry using sterile saline for tract embolization to prevent pneumothorax after lung biopsy.~~Particularly in transportal procedures, radiopaque materials and devices have been used to ensure tract closure [19, 20]. A previous report confirmed closure of the transhepatic route by injecting a slurry made with 1 sheet of Gelfoam and 3 mL of contrast media [21] (Page 21, Line 334 - 335)

~~In both clinical and animal studies,~~In the present study,

**Response:** We have also added References #19, 20, and 21 in the revised manuscript.

Reference #19

Zhang ZH, • Zhang W, Liu QX, Ma JQ, Yang MJ, Liu LX, et al. Embolization of the transhepatic tract after percutaneous portal vein interventions: single-centre retrospective study comparing n-butyl cyanoacrylate versus coils. Cardiovasc Intervent Radiol 2021; 44:1728-1733.

Reference #20

Tan G, Yong E, Sathyanarayana R, Brooks M, Jhamb A. Feasibility of Mynxgrip®-assisted percutaneous transhepatic portal venous access closure. Cardiovasc Intervent Radiol 2020; 43:1938-1941.

Reference #21

Uller W, Müller-Wille R, Grothues D, Schelling J, Zausig N, Loss M, et al. Gelfoam for closure of large percutaneous transhepatic and transsplenic puncture tracts in pediatric patients. Rofo 2014; 186: 693-697.

**R-17. Line 320 – 322: “In tract embolization with conventional embolic materials using gelatin sponge, whether the sponge was completely retained within the tract remained uncertain.”**

**This sentence is incomprehensible in its current form.**

**Response:** Thank you for pointing this out. In this sentence, by “conventional embolic materials” we mean gelatin sponge particles with normal saline and gelatin sponge torpedo as used in previous reports (References 6 and 19). These materials are radiolucent and invisible under X-ray fluoroscopy. Thus, these conventional embolic materials using gelatin sponge were difficult to identify in the tract route under X-ray fluoroscopy during and just after the procedure.

As you mentioned, readers might have misunderstood the meaning of this sentence. We have changed the sentence in the revised manuscript as follows.

(Page 22, Line 340 – 343)

In tract embolization using ~~conventional embolic materials using gelatin sponge~~ gelatin sponge particles with normal saline or gelatin sponge torpedo, whether the sponge was completely retained within the tract remained uncertain under X-ray fluoroscopy.

We have changed the phrasing of the following words considering sentence flow as described below.

(Page 22, Line 344)

such conventional materials using gelatin sponge

**R-18. Lines 322 – 324: “Clinicians have voiced concerns about the displacement of conventional materials from the tract, which might result in tract bleeding.”**

**Does this statement correspond to the internal experiences of the authors or is it based on the reports from the literature? If the latter applies, please cite the corresponding literature.**

**Response:** We have supported the sentence in question with the two following references, which were cited in the Discussion section of the original manuscript.

As pointed out in the response to R-15, we have deleted the sentence,”~~Previous studies have reported that tract embolization using gelatin sponge alone was demonstrated to fail in some cases due to plug malposition or expulsion from the hepatic tract [24,25].~~” We have therefore transferred the two references to the sentence, “Clinicians have voiced concerns about the displacement of conventional materials from the tract, which might result in tract bleeding.”

Please see the sentence and references below.

(Page 22, Line 347 – 349)

~~Previous studies have reported that tract embolization using gelatin sponge alone was demonstrated to fail in some cases due to plug malposition or expulsion from the hepatic tract [24,25].~~

(Page 22, Line 343 – 345)

Clinicians have voiced concerns about the displacement of such conventional materials using gelatin sponge from the tract, which might result in tract bleeding [24,25].

(Reference 24 and 25)

24. Froud T, Yrizarry JM, Alejandro R, Ricordi C. Use of D-STAT to prevent bleeding following percutaneous transhepatic intraportal islet transplantation. Cell Transplant 2004; 13:55–5923.

25. Gaba RC, Kobayashi K, Bui JT, Lokken RP, Lipnik AJ, Ray CE, et al. Liver track embolization after islet cell transplant: comparison of two techniques. Am J Roententgenol 2017; 208:1134-1140.

The two references can be summarized as follows:

Reference #24: Froud et al. presented a method of tract closure using an embolic material called D-statTM added to the distal side of the gelatin sponge placed in the tract to prevent tract bleeding in percutaneous transhepatic islet transplantation. The authors stated that “In our experience, gelatin sponge alone has been used to plug the catheter tract (n = 47); however, in the aforementioned three patients, this technique failed, either due to dislodgement of, or bleeding peripheral to, the plug.” and “gelatin sponge alone can be used but has been demonstrated to fail in some cases due to antegrade movement into a portal or hepatic vein, or due to retrograde movement into the peritoneal cavity, and it can be cumbersome to perform. If the tract is short due to peripheral portal vein access, the catheter can become dislodged with respiratory motion before adequate plugging is achieved. If only a portion of the tract is plugged, bleeding can occur from the peripheral tract.” (Cell Transplantation, 2004; 13:55–59)

Reference #25: Gaba et al. also reported that major tract bleeding after gelatin sponge embolization for transhepatic islet cell transplantation occurred in 4 of 35 cases. The authors mentioned that: “(t)wo such cases (of 4 cases) were related to plug malposition or expulsion from the liver track, occurrences that mirror the experience of Froud et al.” (Am J Roententgenol 2017; 208:1134-1140)

**R-19. Do the authors also have experience with the embolization of transhepatic biliary tract routes utilizing the gel-like embolic agent?**

**Response:** Thank you for this interesting question. We have no experience with embolization of the transhepatic biliary tract route using this gel-like embolic material. We see no reason to believe that our method would not be feasible for biliary tract embolization, and verification of safety and feasibility can be expected in future studies.

**R-20. Line 371 – 372: I don´t really see the point in mentioning in the limitation section that embolization in the surgically exposed animal kidneys was faster than in clinical routine. You might mention this in the results section if appropriate.**

**Response:** We assumed that the procedure time from sheath insertion to the end of tract embolization contributed to the success rate of tract closure, so we have mentioned this in the Limitations section of the revised manuscript. However, procedure time was not evaluated and data were not presented in the Results section, so the original sentence might have confused readers. We have therefore removed this description.

(Page 24, Line 392 – Page 25, Line 393)

~~The procedure time from puncture to tract embolization was about 5 min, which is obviously shorter than the clinical procedure time in humans.~~
