# Supplementary material for: Feasibility of gel-like radiopaque embolic material using gelatin sponge and contrast agent for tract embolization after percutaneous treatment
Source: PLoS One. 2023 Feb 3;18(2):e0281384. doi: 10.1371/journal.pone.0281384 (PMC9897536; doi:10.1371/journal.pone.0281384)
Supplement: Attachment — Submitted filename: ResponseToReviewers - 2023116.doc [file pone.0281384.s007.doc]

**Dear editors and reviewers:**

Thank you for providing us with this opportunity to submit a revised version of our manuscript. We appreciate the comments from the reviewers, which have enabled us to greatly improve our manuscript. We have modified the manuscript based on the feedback provided. We have added the requested material to this revised version of the manuscript as concisely as possible. We believe that the revised manuscript will be of interest to the readers of *PLOS One*.

**Reviewer #1:**

We appreciate your positive comments that our revised version of the manuscript is worthy of publication. We are grateful for the time and energy you expended.

**Reviewer #2:**

Thank you for your kind consideration of our manuscript. We are grateful for your comments, which helped us to improve the manuscript. Please find below our point-by-point responses to the comments provided.

**R2-1. Page 2 Line 28: ‘rabbit kidney’ is not clear, specified it such as ‘rabbit kidney puncture site’.**

**Response:** Thank you for pointing out this oversight. We have revised the phrasing as follows:

(Page 2, line 28)

rabbit kidney puncture site

**R2-2. P3L52: ‘intensive surgical approaches’: invasive or extensive would be appropriate than intensive.**

**Response:** We have revised the phrasing based on your comment.

(Page 3, line 52)

~~intensive~~invasive surgical approaches

**R2-3. P7 table 1. Age: give mean and SD if it is not parametric, give median and ranges.**

**Response:** Thank you for this comment. We have changed the notation to include standard deviation in Table 1.

| **Age, years, mean ± SD ~~(range)~~** | **66 ± 9.3 ~~(43–86)~~** |
| --- | --- |

This change has also been made to age values in Supplementary table 1.

| **Age**, **years**, **mean ± SD ~~(range)~~** | **64.8 ± 12.7 ~~(45-79)~~** |
| --- | --- |

**R2-4. Table 1. PTPE should be more specified, please give numbers of ipsilateral or contralateral access.**

**Response:** Thank you for pointing this out. We have added annotations for the numbers of PTPE. Please see the changes indicated below.

| **Treatment**  **PTPE**  **PTS**  **Portal stenting**  **Other** | **32***  **22**  **1**  **2** |
| --- | --- |

(Page 8, line 109)

* Ipsilateral puncture in 29 cases; contralateral puncture in 4 cases.

This change was made for treatments in Supplementary table 1.

| **Treatment**  **PTPE**  **PTS**  **Portal stenting**  **Other** | **2***  **5**  **2**  **3** |
| --- | --- |

* Ipsilateral puncture in 2 cases.

**R2-5. table 1. Portal branch for percutaneous access route. Does Right mean right main portal vein? Please more specially describe.**

**Response:** Thank you for raising this issue. “Right” in Table 1 refers to the right portal vein before branching into anterior and posterior segmental branches. We have changed the phrasing used and provided more information in Table 1.

| **Portal ~~branch~~vein for percutaneous access route**  **Right portal vein**  **~~Right a~~Anterior branch of right portal vein**  **~~Right p~~Posterior branch of right portal vein**  **Left portal vein** | **3**  **22**  **15**  **17** |
| --- | --- |

These changes in wording have also been applied to Supplementary table 1.

| **Portal ~~branch~~vein for percutaneous access route**  **Right portal vein**  **~~Right a~~Anterior branch of right portal vein**  **~~Right p~~Posterior branch of right portal vein**  **Left portal vein** | **1**  **3**  **1**  **7** |
| --- | --- |

**R2-6. Table 1. Title ‘Postoperative hemorrhage and portal vein thrombosis’ as adverse events.**

**Response:** Thank you for pointing this out. We agree and have provided more information in Table 1.

| **Adverse Events**  **^†^Postoperative hemorrhage**  **No complication**  **Grade 1**  **^†^Portal vein thrombosis**  **No complication**  **Grade 3** | **55 (96.5%)**  **2 (3.5%)**  **55 (96.5%)**   2 **(3.5%)** |
| --- | --- |

Similar changes have been made to Supplementary table 1.

| **Adverse Events**  **^†^Postoperative hemorrhage**  **^†^Portal vein thrombosis** | **N/A**  **N/A** |
| --- | --- |

**R2-7. P9L118 ‘All these squares’ would be better than ‘These squares’.**

**Response:** Thank you for suggesting this improvement. We have changed the phrasing based on this comment.

(Page 8, line 113 – page 9, line 114)

All t~~T~~hese squares

**R-8. P10L148 In response to this line, give mean day and SD of CT obtain in results section.**

**Response:** We agree with your opinion and have provided more information in Table 1 of the revised manuscript.

| **Postoperative days to CT evaluation, days, mean ± SD (range)** | **11.3 ± 8.3 (1-30)** |
| --- | --- |

**R2-9. P19 Fig 3. The phrases “A 7-Fr sheath (black arrowheads)…” are unnecessarily repeated.**

**Response:** Based on this recommendation, we have removed these overused phrases and have simplified the legend for Figure 3 in the revised manuscript.

(Page 19, lines 287 – 291)

b) ~~A 7-Fr sheath (black arrowhead) is inserted into the contrast-enhanced kidney.~~ A coil is inserted in the puncture tract through the 7-Fr sheath (black arrowhead). c) ~~A 7-Fr sheath (black arrowhead) is inserted into the contrast-enhanced kidney.~~ Injection of 0.5 mL of NBCA glue (white arrowhead) through the 7-Fr sheath (black arrowhead) ~~the sheath~~ while withdrawing the sheath.

**R2-10. P21L329 It would be better to note the difference between reference 21 and this study.**

**Response:** Thank you for this comment. Reference #21 confirmed closure of the transhepatic route after injection of a slurry made with 1 sheet of absorbable gelatin sponge (Gelfoam, 2 cm × 6 cm × 0.7 cm; Pfizer, New York, NY, USA) and 3 mL of contrast media. The gel-like material in our study was composed of two sheets of absorbable gelatin sponge (Spongel, 2.5 cm × 5 cm × 1 cm; LTL Pharma, Shinjuku-ku, Tokyo, Japan) and 3 mL of contrast media. The volume of gelatin sponge in the previous study was smaller than that in our study. As a result, the slurry in Reference #21 might have been less viscous than our gel-like embolic material. Based on your suggestion, we have deleted the below sentence from the Discussion section of the revised manuscript.

(Page 20, lines 314 – 316)

~~A previous report confirmed closure of the transhepatic route by injecting a slurry made with 1 sheet of Gelfoam and 3 mL of contrast media [21].~~

We have added the following sentences in the subsequent paragraph of the Discussion section in the revised manuscript.

(Page 21, line 332 – page 22, line 336)

A previous report confirmed closure of the transhepatic route by injecting a slurry made with 1 sheet of Gelfoam and 3 mL of contrast media [25]. The volume of gelatin sponge in the previous study was smaller than that in our study. Our gel-like embolic material might have been more viscous material than the material used in the previous study.

**R2-11. The actual videos were not matched with S1 and S2 video legends. S2 is making video and S1 is procedure.**

**Response:** Thank you for noticing these inconsistencies. We have corrected the name of the mp4 files. S1 is the video of making the embolic material and S2 is the video of procedure”. No changes in numbers have been made in the manuscript.
